# Highly Efficient Flexible Polymer Solar Cells with Robust Mechanical Stability

**DOI:** 10.1002/advs.201801180

**Published:** 2019-02-07

**Authors:** Licheng Tan, Yilin Wang, Jingwen Zhang, Shuqin Xiao, Huanyu Zhou, Yaowen Li, Yiwang Chen, Yongfang Li

**Affiliations:** ^1^ College of Chemistry Nanchang University 999 Xuefu Avenue Nanchang 330031 China; ^2^ Institute of Polymers and Energy Chemistry Nanchang University 999 Xuefu Avenue Nanchang 330031 China; ^3^ Laboratory of Advanced Optoelectronic Materials College of Chemistry Chemical Engineering and Materials Science Soochow University 199 Ren'ai Road Suzhou 215123 China

**Keywords:** bendability, electron transporting layers, flexibility, flexible polymer solar cells

## Abstract

Landmark power conversion efficiency (PCE) over 14% has been accomplished for single‐junction polymer solar cells (PSCs). However, the inevitable fracture of inorganic transporting layers and deficient interlayer adhesion are critical challenges to achieving the goal of flexible PSCs. Here, a bendable and thickness‐insensitive Al‐doped ZnO (AZO) modified by polydopamine (PDA) has emerged as a promising electron transporting layer (ETL) in PSCs. It has special ductility and adhesion to the active layer for improving the mechanical durability of the device. Nonfullerenes PSCs based on PBDB‐T‐2F:IT‐4F with AZO:1.5% PDA (80 nm) ETL yield the best PCE of 12.7%. More importantly, a prominent PCE, approaching 11.5%, is reached for the fully flexible device based on Ag‐mesh flexible electrode, and the device retains >91% of its initial PCE after bending for 1500 cycles. Such thickness insensitivity, mechanical durability, and interfacial adhesion properties for the inorganic ETLs are desired for the development of flexible and wearable PSCs with reliable photovoltaic performance and large‐area roll‐to‐roll printing manufacture.

Polymer solar cells (PSCs), as an attractive alternative for the development of sustainable photovoltaic technology, have been investigated in depth as they can be fabricated by large‐area solution processing while maintaining the features of light weight, flexibility, tunable transparency, etc.^1^, ^2^ Till now, PSCs have achieved power conversion efficiencies (PCEs) of >14% in laboratory scale.^3^, ^4^ In order to realize the industrialization of large‐area flexible and wearable PSCs, individual electronic components in the whole device must possess characteristics including flexibility, long‐term stability, and low cost. Other than those developing stretchable transparent conductive electrodes,^5^, ^6^, ^7^, ^8^ many reports have concentrated on preparing active layers and interfacial layers with superior optoelectronic properties, thickness‐insensitivity, and mechanical properties,^9^, ^10^, ^11^, ^12^, ^13^, ^14^, ^15^, ^16^ as well as large‐area manufacture technique^17^, ^18^, ^19^, ^20^ and device stability evaluation.^21^, ^22^

In contrast to poly(3,4‐ethylenedioxythiophene):poly(styrenesulfonate) (PEDOT:PSS) hole transporting layer (HTL) with hygroscopicity and acidity,^23^ as well as fullerene derivative electron transporting layer (ETL) with aggregation drawback,^24^ inverted PSCs based on metal oxide transporting layer have been proposed to improve the long‐term stability. Zinc oxide (ZnO) is one of the best choices for metal oxide ETLs due to its promising features such as low cost, facile access, nontoxicity, high stability, and unique optical/electronic properties.^25^ However, the widely used sol–gel‐derived ZnO has surface defects ascribed to interstitial zinc and adsorbed oxygen,^26^ and relatively poor interfacial contact with the active layer.^27^ Additionally, pure ZnO is not appropriate to form a thick layer due to its relatively low conductivity, which is not favorable for large‐scale roll‐to‐roll printing. Many strategies, such as aluminum‐doped ZnO (AZO), have been developed to passivate the surface defects and improve the conductivity of ZnO.^28^, ^29^, ^30^ In our previous work, highly conductive and surfactant‐stable AZO nanoparticles (NPs) were prepared by colloidal synthesis procedure with the assistant stabilization by amine surfactants, which afford excellent storage stability and device efficiency with a thick interlayer of >80 nm.^31^

Unfortunately, the inherent brittleness and low adhesion of the inorganic AZO still limits its practical application in flexible and wearable PSCs. This is because the inevitable existence of AZO ETL cracking and AZO/active layer interfacial delamination upon blending can seriously deteriorate the charge transportation and extraction in PSCs. However, to our knowledge, there is no report regarding this important topic. Facchetti and co‐workers reported that the ultraflexible and transparent metal oxide transistors can be realized by doping insulating polymer polyvinylphenole (PVP) into indium oxide (In_2_O_3_) precursor formulation solution and processing at 225 °C to achieve polycrystalline films.^32^ However, the high thermal annealing temperature limits its application in flexible PSCs due to the low glass transition temperature of plastic substrate, such as polyethylene terephthalate (PET). Moreover, the high doping content of PVP would greatly deteriorate intrinsic long‐term stability of inorganic materials. Nevertheless, this method raises the hope of using inorganic transporting layer in flexible PSCs toward robust mechanical properties.

Herein, novel bendable AZO derivatives were exploited for using as ETLs in fully flexible PSCs, which have been fabricated through in situ approach by incorporating low‐content polydopamine (PDA) into AZO. We find that PDA can effectively passivate surface defect sites of AZO through formation of strong intermolecular hydrogen‐bonding interactions. The PDA‐modified AZO exhibit high electron mobility under low temperature process and excellent mechanical properties in terms of flexural endurance and interfacial adhesion to the upper organic active layer. Nonfullerene PSCs based on PBDB‐T‐2F:IT‐4F with AZO:1.5% PDA (80 nm) ETL yield the best PCE of 12.7%. Furthermore, a prominent PCE, approaching 11.5%, has been accomplished for the fully flexible PSCs. To our best knowledge, this is the highest efficiency among reported flexible PSCs.^33^, ^34^, ^35^, ^36^, ^37^, ^38^, ^39^ Importantly, the flexible device can retain >91% of its initial PCE after 1500 bending cycles. The well‐formed active layer on AZO:1.5% PDA is barely peeled off even after 3M tape peeling 10 times, indicating the existence of excellent interlayer adhesion. These excellent mechanical properties of the modified AZO ETLs afford a new opportunity in fully flexible PSCs. Moreover, the thickness‐insensitivity (with thickness up to ≈80 nm) and long‐term stability make it more beneficial for the large‐area processing technique in mass production of photovoltaic modules.

In order to explore the effect of incorporating insulated PDA, transmittance and photoluminescence (PL) measurements have been conducted to investigate the optical properties of AZO and the PDA‐modified AZO. As shown in **Figure** **1**a, all the AZO films with different amounts of PDA exhibit enhanced transparencies (>90%) in the wavelength range of 400–800 nm compared with that of the unmodified AZO, providing the feasibility for its application as ETLs in the inverted PSCs. From PL spectra in Figure 1b, two emission peaks are observed for all the samples. The broader one, at 400–550 nm, is assigned to the deep level emission (DLE), which is caused by defect sites including oxygen vacancies and zinc interstitial.^40^ In contrast, the PDA‐modified AZO shows much lower DLE peak intensity than that of AZO, indicating a lower concentration of deep‐level defects. This behavior can be further demonstrated by the high‐resolution X‐ray photoelectron spectroscopy (XPS) spectra measurements. As shown in Figure 1c, the O 1s XPS spectra show two typical peaks, at about 530.1 and 531.5 eV, which are assigned to O^2−^ ions in Zn—O bonds and oxygen deficiency, respectively. As for PDA‐modified AZO, a distinct decline in the intensity of the second peak at 531.5 eV confirms that defects and traps of AZO have been effectively passivated by the PDA.^41^ This passivation effect could be explained by the interaction between AZO and PDA through the N—Zn bond, as evidenced from slight peak position shift of N 1s toward lower binding energy value in the XPS spectra of AZO and the PDA‐modified AZO (Figure S1, Supporting Information). In addition, Figure S2 in the Supporting Information depicts the Zn 2p XPS spectra of AZO and the PDA‐modified AZO. The peak position of Zn 2p_3/2_ for the PDA‐modified AZO shifting toward a higher binding energy again demonstrates the interaction between AZO and PDA through the N—Zn bond.^42^ Ultraviolet photoelectron spectroscopy (UPS) measurement (Figure S3, Supporting Information) has also been used to study the energy levels of AZO and the PDA‐modified AZO. As seen from Figure 1d, the corresponding conduction bands for AZO, AZO:1.0% PDA, AZO:1.5% PDA, and AZO:2.0% PDA are determined as −4.30, −4.24, −4.11, and −4.17 eV, respectively. For AZO:1.5% PDA, there is more favorable band alignment with the lowest unoccupied molecular orbital (LUMO) of the acceptors, which is consistent with the increased photocurrent when using AZO:1.5% PDA as ETL in PSCs. Meanwhile, a typical wurtzite crystal structure^30^ is determined for AZO and the PDA‐modified AZO by X‐ray diffraction (XRD) patterns measurement (Figure S4, Supporting Information), which shows that the addition of PDA has no influence on the crystallinity of AZO. These results demonstrate that the PDA‐incorporated AZO not only has lower defect concentration, but also facilitates to form Ohm contact with active layer, leading it to serving as an ideal ETL to improve device performance.

**Figure 1:**
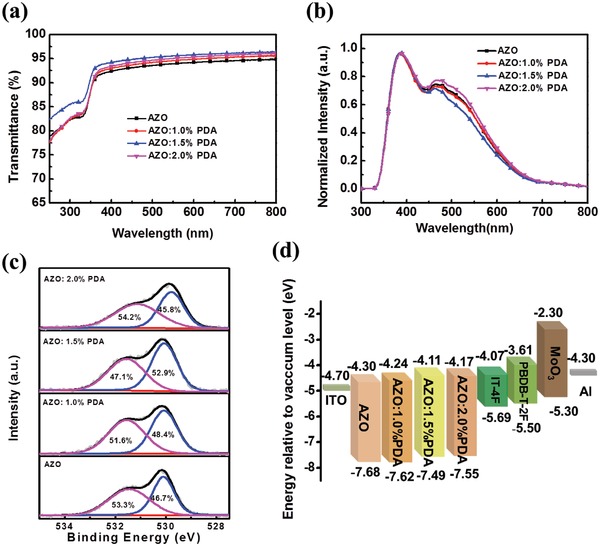
The impact of additive PDA (with different molar fractions) on the basic performance of AZO: a) Transmittance spectra, b) room temperature PL spectra excited at 315 nm, c) O 1s XPS spectra, and d) energy levels.

To the best of our knowledge, most of the highly efficient PSCs are fabricated based on very thin ETLs (≈2–10 nm) due to its relatively low conductivity, which is highly problematic in high‐throughput processing methods. Accordingly, increasing attention has been paid recently to the development of new ETL materials with high charge mobility,^42^ so that they could work efficiently under thicker ETLs. In this work, we demonstrate AZO and the PDA‐modified AZO to be thickness‐insensitive ETLs by conducting the electron mobility measurement on thick film (≈80 nm). It should be noted that both the AZO and PDA‐modified AZO films were processed under low temperature of 140 °C for 20 min. In atomic force microscopy (AFM) images (Figure S5, Supporting Information), thick AZO films present unique nanoripple texture, showing a rougher surface than that of the corresponding thin films. This feature can provide a larger contact area when contacting with active layer, hence improving charge transfer and collection efficiency.^43^ To better understand the effect of PDA on the electron transport properties of AZO ETLs, electron‐only devices have been fabricated with two device configurations, in which ITO/AZO or AZO:PDA ETLs/PBDB‐T:ITIC/Al configuration is used to reveal the electron mobility of the whole devices (Figure S6a, Supporting Information), while the ITO/AZO or AZO:PDA ETLs/Al are used to test the conductivities of AZO and the modified AZO (Figure S6b, Supporting Information). The effective electron mobility (*µ*_e_) of AZO and the PDA‐modified AZO ETLs in electron‐only devices has been estimated by using space‐charge‐limited‐current (SCLC) method on the basis of the Mott–Gurney equation,^44^ as summarized in Table S1 in the Supporting Information. With thick interlayer (80 nm), the devices display one order of magnitude higher *µ*_e_, based on AZO:1.5% PDA (4.2 × 10^−3^ cm^2^ V^−1^ s^−1^), compared to the corresponding AZO‐based devices (6.1 × 10^−4^ cm^2^ V^−1^ s^−1^). The same variation has also been observed from the conductivity values, as summarized in Table S2 in the Supporting Information, which is tentatively ascribed to defect passivation of the PDA‐modified AZO.

First, the photovoltaic performances of the PBDB‐T‐2F:IT‐4F, PBDB‐T:ITIC,and PTB7‐Th:PC_71_BM‐based inverted PSCs (the molecular structures of the active layer materials are shown in Figure S7 in the Supporting Information) with AZO and PDA‐modified AZO ETLs were investigated. The current density–voltage (*J*–*V*) curves and incident photon‐to‐current efficiency (IPCE) spectra are given in Figure S8 in the Supporting Information, while the detailed photovoltaic characteristics are listed in Table S3 in the Supporting Information. The PSCs based on PBDB‐T:ITIC with AZO:1.5% PDA (80 nm) yield higher PCE of 10.3%, with a short circuit current density (*J*_sc_) of 17.68 mA cm^−2^, an open‐circuit voltage (*V*_oc_) of 0.886 V, and a fill factor (FF) of 65.3%. The integrated *J*_sc_ values from IPCE spectra are 16.01, 16.60, 16.93, and 16.31 mA cm^−2^ for the devices with the ETLs of AZO, AZO:1.0% PDA, AZO:1.5% PDA, and AZO:2.0% PDA, respectively, which are well in agreement with those obtained from *J*–*V* curves (≈4% mismatch). Then, the long‐term stabilities of the inverted PSCs based on PBDB‐T:ITIC and AZO or AZO:PDA ETLs were evaluated. The PCE degradation as a function of storage time in nitrogen‐filled glove box is shown in Figure S9 in the Supporting Information. The unencapsulated PSCs based on AZO:1.5% PDA ETL exhibit superior stability and maintain >85% of the original PCE value after storage for 30 d. Such pre‐eminent stability can be attributed to excellent interfacial interaction between AZO:PDA and active layer. Moreover, in order to demonstrate universality of the PDA modified AZO ETL, PBDB‐T‐2F:IT‐4F active layer was used to fabricate inverted PSCs on both glass and plastic substrate. As shown in **Figure** **2**a and **Table** **1**, the best PCE of 12.7%, with *J*_sc_ of 21.74 mA cm^−2^, *V*_oc_ of 0.83 V, and FF of 70.4%, is achieved by employing AZO:1.5% PDA ETL on ITO glass substrate. This PCE value is higher than that of the PSCs device with AZO ETL (12.3%). The integrated *J*_sc_ values from IPCE spectra are 20.75 and 20.81 mA cm^−2^ for AZO and AZO:1.5% PDA (Figure 2b), which agrees well with those from *J*–*V* curves. When we transferred AZO and AZO:1.5% PDA ETLs to PET/Ag mesh–based flexible electrode, a high PCE of 11.5% can be still obtained with a *J*_sc_ of 19.97 mA cm^−2^, a *V*_oc_ of 0.82 V, and a FF of 70.3% (Figure 2c) for the AZO:1.5% PDA‐based flexible device. The 11.5% PCE represents the highest PCE value for the flexible PSCs reported to date owing to the AZO:1.5% PDA ETL with thickness‐insensitive and bendable properties. To verify the bendability of AZO:PDA, the normalized PCEs (Figure 2d) of the corresponding device as a function of bending cycles with radius of 6 mm were measured. The AZO:1.5% PDA‐based fully flexible device retains >91% of its initial PCE after 1500 bending cycles. The similar result could also be observed in the PBDB‐T:ITIC‐based flexible PSCs (Figure S10, Supporting Information). Furthermore, the specific device parameters based on flexible polymer solar cells after bending test are summarized in Table S4 in the Supporting Information. Such pre‐eminent mechanical properties of flexible devices are mainly attributed to the incorporation of PDA into AZO, which effectively improves the bending durability of AZO:PDA ETL and enhances interfacial interaction with active layer upon the bending process.

**Figure 2:**
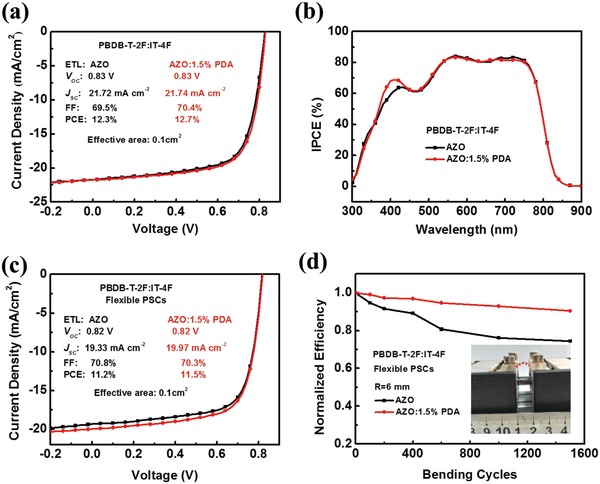
Basic device characteristics: a) *J*–*V* curves and b) IPCE spectra of inverted PSCs based on ITO/AZO ETLs/PBDB‐T‐2F:IT‐4F/MoO_3_/Al under simulated 100 mW cm^−2^ AM 1.5 G illumination. c) *J*–*V* curves of flexible devices based on PET/Ag‐mesh/PH1000/PEIE/AZO ETLs/PBDB‐T‐2F:IT‐4F/MoO_3_/Al. d) Normalized average PCE of flexible PSCs as a function of bending cycles with radius of 6 mm.

**Table 1:** Device parameters of inverted PSCs based on PBDB‐T‐2F:IT‐4F and PBDB‐T:ITIC active layer with AZO and bendable AZO:1.5% PDA ETLs under AM 1.5 G irradiation (100 mW cm^−2^). The device effective area is 0.1 cm^2^ of a single chip; all the values represent averages from at least 12 devices on a single chip.The normal device structure is ITO/AZO ETLs/Active layer/MoO_3_/Al and the flexible device structure is PET/Ag‐mesh/PH1000/PEIE/AZO ETLs/Active layer/MoO_3_/Al

Device type	BHJ	*J*_sc_ a) [mA cm^−2^]	*V*_oc_ [V]	FF [%]	PCE [%]
AZO‐PSCs	PBDB‐T‐2F:IT‐4F	21.61 ± 0.11 [20.75]b)	0.830 ± 0.002	69.2 ± 0.3	12.1 ± 0.2 [12.3]a)
AZO:1.5% PDA‐PSCs	PBDB‐T‐2F:IT‐4F	21.62 ± 0.12 [20.81]b)	0.830 ± 0.004	70.1 ± 0.3	12.5 ± 0.2 [12.7]a)
AZO‐flexible PSCs	PBDB‐T‐2F:IT‐4F	19.15 ± 0.18	0.820 ± 0.003	70.6 ± 0.2	11.0 ± 0.2 [11.2]a)
AZO:1.5% PDA‐flexible PSCs	PBDB‐T‐2F:IT‐4F	19.82 ± 0.15	0.820 ± 0.003	70.0 ± 0.3	11.3 ± 0.2 [11.5]a)
AZO‐flexible PSCs	PBDB‐T:ITIC	14.89 ± 0.19	0.89 ± 0.002	67.9 ± 0.3	9.0 ± 0.2 [9.2]a)
AZO:1.5% PDA‐flexible PSCs	PBDB‐T:ITIC	16.09 ± 0.16	0.89 ± 0.002	66.9 ± 0.2	9.4 ± 0.2 [9.6]a)

^a)^ The best PCE value

^b)^ Values are calculated from IPCE.

It is well known that the inherent brittleness of the inorganic AZO limits its practical application in flexible and wearable PSCs. In this work, an important challenge for achieving bendability of AZO ETLs has been addressed for the first time. **Figure** **3**a–l shows scanning electron microscope (SEM) images of unmodified AZO, AZO:1.5% PDA, AZO/PBDB‐T:ITIC, and AZO:1.5% PDA/PBDB‐T:ITIC films before and after bending for 50 or 200 cycles. Evident fractures are observed in both AZO and AZO/PBDB‐T:ITIC films upon bending for 50 cycles, while the AZO:1.5% PDA and AZO:1.5% PDA/PBDB‐T:ITIC can well maintain the initial uniform morphology even after 200 bending cycles. This result indicates that PDA can significantly increase the mechanical stability of AZO in terms of bending, because of the flexible PDA acting as an elastomer binder among AZO nanocrystals. To further quantify the effect of PDA on the flexural endurance of AZO, the AFM peak‐force model was carried out to analyze the Young's modulus. As shown in Figure S11 in the Supporting Information, the AZO:1.5% PDA presents a Young's modulus value of 127 MPa, which is much lower than that of the pure AZO (245 MPa), demonstrating a better ductility of AZO:PDA composite. In addition, there is no obvious variation on transmittance for both AZO and AZO:1.5% PDA before and after bending (Figure 3m). It is worth mentioning that the PDA‐elasticized AZO shows higher transparency in contrast with pristine AZO, which might be attributed to depression of defects and improvement of uniformity of heterogeneous nanocrystals. However, more distinct reduction of UV–vis absorption intensity for PBDB‐T:ITIC active layer on AZO after bending for 50 cycles has been noticed, in comparison to that on bendable AZO:1.5% PDA, as shown in Figure 3n. These results confirm that the incorporation of a lesser amount of elastomer PDA additives could release mechanical stress of active layer under the flexural experiences by assistance of durable inorganic AZO.

**Figure 3:**
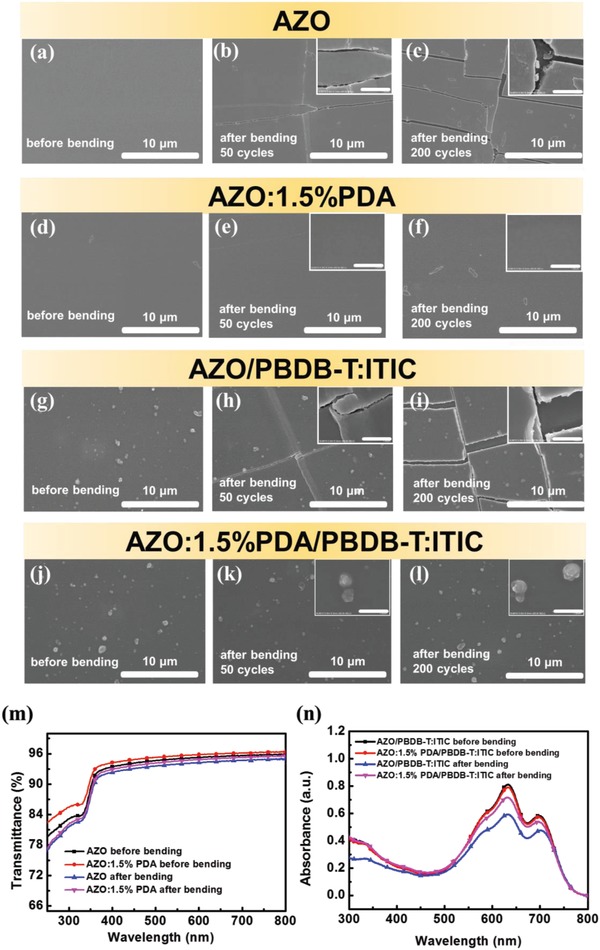
Bending performance: SEM images of a–c) AZO, d–f) bendable AZO:1.5% PDA, g–i) AZO/PBDB‐T:ITIC, and j–l) AZO:1.5% PDA/PBDB‐T:ITIC on PET substrate before and after bending. Insets show the corresponding magnified SEM images, and the scale bar is 1 µm. m) Transmittance spectra of AZO and AZO:1.5% PDA on PET substrate before and after bending for 50 cycles. n) UV–vis absorption spectra of AZO/PBDB‐T:ITIC and AZO:1.5% PDA/PBDB‐T:ITIC on PET substrate before and after bending for 50 cycles.

Another key factor affecting mechanical properties of PSCs is the adhesion property between functional layers in device. In this work, we carried out the peeling process via 3M tape to test the interfacial adhesion, which is similar to the approach that used for peeling perovskite layer from ZnO with scotch tape.^45^ As shown in **Figure** **4**a and Movies S1–S4 in the Supporting Information, we first applied a force of 0.5 MPa to stick 3 M tape with active layer by a tablet machine. Subsequently, the sample is put on a universal testing machine for peeling test (2 N peeling force, 50 mm min^−1^ peeling speed and 180° peeling direction). Obviously, the active layer was easily detached from the unmodified AZO film after peeling 5 times, which is due to the poor adhesion between active layer and AZO. The well‐formed active layer on AZO:1.5% PDA is barely peeled off even after 10 times, due to the excellent interfacial adhesion promoted by strong intermolecular interactions between the functional groups of PDA and organic active layer. Furthermore, UV–vis absorption measurement has been used to verify the absorption intensity variation of the active layer before and after the peeling process. As seen from Figure 4b,c, there is no obvious reduction on absorption intensity of active layer based on the AZO:1.5% PDA ETL, with a comparison to that based on the unmodified AZO, which suggests that the active layer retains uniformity and integrality due to existence of strongly adhesive PDA‐modified AZO under peeling process. The enhanced adhesion property and mechanical property of devices could be attributed to the strong intermolecular interactions between PDA and organic active layer. To confirm the mechanism of interaction, the Fourier transform infrared spectroscopy (FTIR) measurement was conducted. Note that the large‐content PDA was blended with PTB7‐Th:PC_71_BM for clearly observing intermolecular interaction. As shown in **Figure** **5**a, the peaks in the range of 3000–3500 cm^−1^ and at ≈1750 cm^−1^ was assigned to the symmetric stretching vibrations of —NH and —OH (ν(N—H) and ν (O—H)), and —C=O (ν(C=O)) in PDA, respectively. The PTB7‐Th:PC_71_BM:PDA blend film showed a stronger and wider ν(N—H) and ν(O—H) than those of pure PDA film indicating the formed hydrogen bond between PDA and PTB7‐Th/PC_71_BM. This behavior was further confirmed by the shifted symmetric stretching vibrations of —C=O (ν(C=O)) toward short wavenumber, which can also be attributed to the formed hydrogen bond.^46^, ^47^ Similar mechanism was also demonstrated in PBDB‐T:ITIC:PDA and PBDB‐T‐2F:IT‐4F:PDA active layer systems (Figure S12, Supporting Information). Therefore, as shown in Figure 5b, the —NH and —OH groups of PDA in AZO:PDA interfacial layer can form hydrogen bonds with the —O and —C=O of PTB7‐Th:PC_71_BM, which could enhance interaction between active layer and interfacial layer, thus improving the mechanical adhesion and durability of the resulting devices. These excellent characteristics including bendability and interfacial adhesion of the PDA‐elasticized AZO ETLs offer a new opportunity to realize the goal of flexible and wearable PSCs with reliable performance.

**Figure 4:**
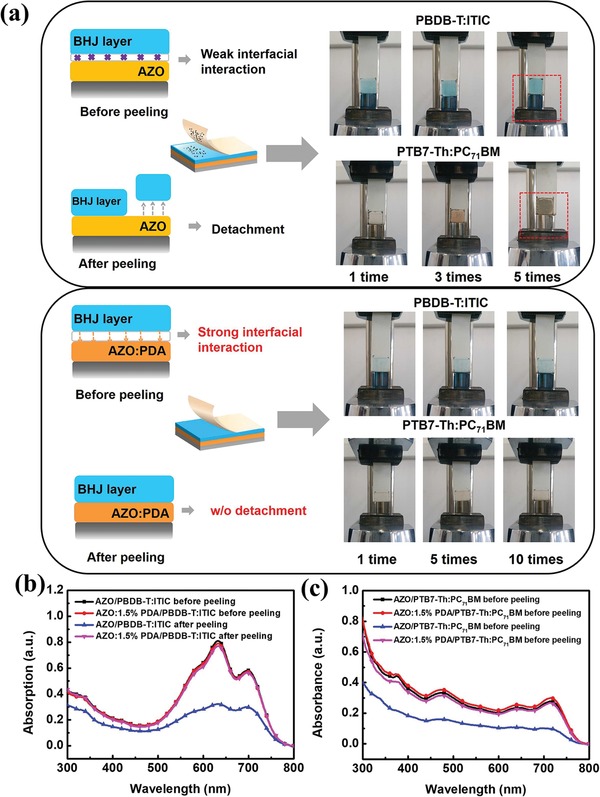
Adhesion performance: a) Photographs of interfacial adhesion by 3 mtape peeling test. b,c) UV–vis absorption spectra of AZO/PBDB‐T:ITIC, AZO:1.5% PDA/PBDB‐T:ITIC, AZO/PTB7‐Th:PC_71_BM, and AZO:1.5%PDA/PTB7‐Th:PC_71_BM before and after peeling 5 times.

**Figure 5:**
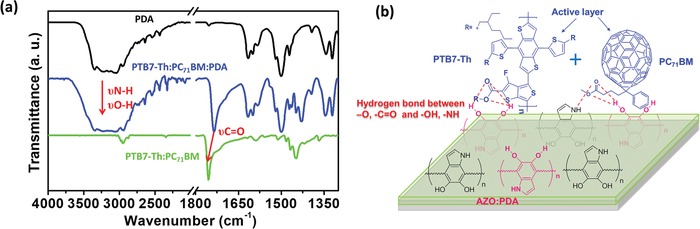
a) FTIR spectra of PDA, PTB7‐Th:PC_71_BM:PDA and PTB7‐Th:PC_71_BM. b) Schematic illustration of the mechanism of enhanced mechanical adhesion and durability for the AZO:PDA interfacial layer–based device.

In conclusion, a bendable and thickness‐insensitive ETL with adhesiveness has been in situ fabricated by introducing PDA into AZO as cross‐linking agent among nanocrystals. The PDA‐incorporated AZO exhibits superiority in the following several aspects: a) superior optoelectronic properties with high transparency and conductivity, which can be applied as thickness‐insensitive ETL; b) enhancing the flexural endurance of inorganic ETL upon bending, which is beneficial to the flexibility of the PSC devices; and c) endowing strong interfacial adhesion between organic active layer and inorganic ETL, which is in favor of enhancement of device durability. The best PCE of 12.7%, based on the PBDB‐T‐2F:IT‐4F system, has been achieved by employing the AZO:1.5% PDA ETL with thickness of 80 nm, which is superior to PCE of the devices with such thick interlayer reported to date. More importantly, a champion PCE of 11.5% has been obtained for fully flexible PSCs based on PET/Ag‐mesh electrode, and the device retains >91% of its initial PCE after bending for 1500 cycles. Such excellent mechanical durability and thickness‐insensitivity of inorganic ETLs are beneficial to roll‐to‐roll fabrication of large‐area flexible and wearable PSCs.

## Conflict of Interest

The authors declare no conflict of interest.

## Supporting information

Supplementary

Supplementary

Supplementary

Supplementary

Supplementary

## References

[1] B. C. Thompson , J. M. J. Fréchet , Angew. Chem., Int. Ed. 2008, 47, 58. 10.1002/anie.200702506 18041798

[2] V. Shrotriya , Nat. Photonics 2009, 3, 447.

[3] S. Q. Zhang , Y. P. Qin , J. Zhu , J. H. Hou , Adv. Mater. 2018, 30, 1800868. 10.1002/adma.201800868 29602243

[4] S. S. Li , L. Ye , W. C. Zhao , H. P. Yan , B. Yang , D. L. Liu , W. N. Li , H. Ade , J. H. Hou , J. Am. Chem. Soc. 2018, 140, 7159. 29737160 10.1021/jacs.8b02695

[5] X. Yang , X. T. Hu , Q. X. Wang , J. Xiong , H. J. Yang , X. C. Meng , L. C. Tan , L. Chen , Y. W. Chen , ACS Appl. Mater. Interfaces 2017, 9, 26468. 28731322 10.1021/acsami.7b08606

[6] W. X. Guo , Z. J. Xu , F. Y. Zhang , S. Y. Xie , H. Y. Xu , X. Y. Liu , Adv. Funct. Mater. 2016, 26, 8855.

[7] B. N. Chandrashekar , B. Deng , A. S. Smitha , Y. B. Chen , C. W. Tan , H. X. Zhang , H. L. Peng , Z. F. Liu , Adv. Mater. 2015, 27, 5210. 26256002 10.1002/adma.201502560

[8] G. Y. Xu , L. Shen , C. H. Cui , S. P. Wen , R. M. Xue , W. J. Chen , H. Y. Chen , J. W. Zhang , H. K. Li , Y. W. Li , Y. F. Li , Adv. Funct. Mater. 2017, 27, 1605908.

[9] Z. H. Chen , P. Cai , J. W. Chen , X. C. Liu , L. J. Zhang , L. F. Lan , J. B. Peng , Y. G. Ma , Y. Cao , Adv. Mater. 2014, 26, 2586. 24488944 10.1002/adma.201305092

[10] X. Y. Liu , L. Ye , W. C. Zhao , S. Q. Zhang , S. S. Li , G. M. Su , C. Wang , H. Ade , J. H. Hou , Mater. Chem. Front. 2017, 1, 2057.

[11] Y. Cui , B. W. Xu , B. Yang , H. F. Yao , S. S. Li , J. H, Hou , Macromolecules 2016, 49, 8126.

[12] Z. G. Zhang , B. Y. Qi , Z. W. Jin , D. Chi , Z. Qi , Y. F. Li , J. Z. Wang , Energy Environ. Sci. 2014, 7, 1966.

[13] J. W. Zhang , R. M. Xue , G. Y. Xu , W. J. Chen , G. Q. Bian , C. A. Wei , Y. W. Li , Y. F. Li , Adv. Funct. Mater. 2018, 28, 1705847.

[14] C. Lu , W. Y. Lee , X. D. Gu , J. Xu , H. H. Chou , H. P. Yan , Y. C. Chiu , M. Q. He , J. R. Matthews , W. J. Niu , J. B. H. Tok , M. F. Toney , W. C. Chen , Z. N. Bao , Adv. Electron. Mater. 2017, 3, 1600311.

[15] T. Sekitani , Y. Noguchi , K. Hata , T. Fukushima , T. Aida , T. Someya , Science 2008, 321, 1468. 18687922 10.1126/science.1160309

[16] Z. H. Wu , C. Sun , S. Dong , X. F. Jiang , S. P. Wu , H. B. Wu , H. L. Yip , F. Huang , Y. Cao , J. Am. Chem. Soc. 2016, 138, 2004. 26794827 10.1021/jacs.5b12664

[17] T. R. Andersen , H. F. Dam , M. Hösel , M. Helgesen , J. E. Carlé , T. T. Larsen‐Olsen , S. A. Gevorgyan , J. W. Andreasen , J. Adams , N. Li , F. Machui , G. D. Spyropoulos , T. Ameri , N. Lemaître , M. Legros , A. Scheel , D. Gaiser , K. Kreul , S. Berny , O. R. Lozman , S. Nordman , M. Välimäki , M. Vilkman , R. R. Søndergaard , M. Jørgensen , C. J. Brabec , F. C. Krebs , Energy Environ. Sci. 2014, 7, 2925.

[18] S. Berny , N. Blouin , A. Distler , H. J. Egelhaaf , M. Krompiec , A. Lohr , O. R. Lozman , G. E. Morse , L. Nanson , A. Pron , T. Sauermann , N. Seidler , S. Tierney , P. Tiwana , M. Wagner , H. Wilson , Adv. Sci. 2016, 3, 1500342. 10.1002/advs.201500342 PMC5064630 27774403

[19] S. Hong , H. Kang , G. Kim , S. Lee , S. Kim , J. H. Lee , J. Lee , M. J. Yi , J. Kim , H. Back , J. Kim , K. Lee , Nat. Commun. 2016, 7, 10279. 26728507 10.1038/ncomms10279 PMC4728442

[20] L. Zhang , B. J. Lin , B. Hu , X. B. Xu , W. Ma , Adv. Mater. 2018, 30, 1800343. 10.1002/adma.201800343 29665119

[21] S. X. Li , L. L. Zhan , F. Liu , J. Ren , M. M. Shi , C. Z. Li , T. P. Russell , H. Z. Chen , Adv. Mater. 2018, 30, 1705208. 10.1002/adma.201705208 29271518

[22] D. Baran , R. S. Ashraf , D. A. Hanifi , M. Abdelsamie , N. Gasparini , J. A. Röhr , S. Holliday , A. Wadsworth , S. Lockett , M. Neophytou , C. J. M. Emmott , J. Nelson , C. J. Brabec , A. Amassian , A. Salleo , T. Kirchartz , J. R. Durrant , I. McCulloch , Nat. Mater. 2017, 16, 363. 27869824 10.1038/nmat4797

[23] M. P. de Jong , L. J. van Ijzendoorn , M. J. A. de Voigt , Appl. Phys. Lett. 2000, 77, 2255.

[24] G. Angelini , P. D. Maria , A. Fontana , M. Pierini , Langmuir 2001, 17, 6404.

[25] J. Huang , Z. G. Yin , Q. D. Zheng , Energy Environ. Sci. 2011, 4, 3861.

[26] S. Mahamuni , B. S. Bendre , V. J. Leppert , C. A. Smith , D. Cooke , S. H. Risbud , H. W. H. Lee , Nanostruct. Mater. 1996, 7, 659.

[27] L. E. Greene , M. Law , B. D. Yuhas , P. D. Yang , J. Phys. Chem. C 2007, 111, 18451.

[28] M. Gaceur , S. B. Dkhi , D. Duché , F. Bencheikh , J. J. Simon , L. Escoubas , M. Mansour , A. Guerrero , G. Garcia‐Belmonte , X. J. Liu , M. Fahlman , W. Dachraoui , A. K. Diallo , C. Videlot‐Ackermann , O. Margeat , J. Ackermann , Adv. Funct. Mater. 2016, 26, 243.

[29] L. K. Jagadamma , M. Al‐Senani , A. El‐Labban , I. Gereige , G. O. Ngongang Ndjawa , J. C. D. Faria , T. Kim , K. Zhao , F. Cruciani , D. H. Anjum , M. A. McLachlan , P. M. Beaujuge , A. Amassian , Adv. Energy Mater. 2015, 5, 1500204.

[30] X. H. Liu , X. D. Li , Y. R. Li , C. J. Song , L. P. Zhu , W. J. Zhang , H. Q. Wang , J. F. Fang , Adv. Mater. 2016, 28, 7405. 27309840 10.1002/adma.201601814

[31] Y. L. Wang , Z. Y. Peng , S. Q. Xiao , J. Yang , H. Y. Zhou , L. Q. Huang , L. L. Sun , Y. H. Zhou , L. C. Tan , Y. W. Chen , Sci. China: Chem. 2018, 61, 127.

[32] X. G. Yu , L. Zeng , N. J. Zhou , P. J. Guo , F. Y. Shi , D. B. Buchholz , Q. Ma , J. S. Yu , V. P. Dravid , R. P. H. Chang , M. Bedzyk , T. J. Marks , A. Facchetti , Adv. Mater. 2015, 27, 2390. 25712894 10.1002/adma.201405400

[33] Y. W. Li , G. Y. Xu , C. H. Cui , Y. F. Li , Adv. Energy Mater. 2018, 8, 1701791.

[34] J. W. Zhang , R. M. Xue , G. Y. Xu , W. J. Chen , G. Q. Bian , C. A. Wei , Y. W. Li , Y. F. Li , Adv. Funct. Mater. 2018, 28, 1705847.

[35] K. Yao , X. K. Xin , C. C. Chueh , K. S. Chen , Y. X. Xu , A. K. Y. Jen , Adv. Funct. Mater. 2015, 25, 567.

[36] T. Kim , J. H. Kim , T. E. Kang , C. Lee , H. Kang , M. Shin , C. Wang , B. Ma , U. Jeong , T. S. Kim , B. J. Kim , Nat. Commun. 2015, 6, 8547. 26449658 10.1038/ncomms9547 PMC4633811

[37] L. J. Zuo , S. H. Zhang , M. M. Shi , H. Y. Li , H. Z. Chen , Mater. Chem. Front. 2017, 1, 304.

[38] L. J. Zuo , S. H. Zhang , H. Y. Li , H. Z. Chen , Adv. Mater. 2015, 27, 6983. 26422198 10.1002/adma.201502827

[39] W. Q. Liu , S. Y. Liu , N. K. Zawacka , T. R. Andersen , P. Cheng , X. W. Zhan , F. C. Krebs , H. Z. Chen , J. Mater. Chem. A 2014, 2, 19809.

[40] M. Liu , A. H. Kitai , P. Mascher , J. Lumin. 1992, 54, 35.

[41] D. Q. Gao , J. Zhang , G. J. Yang , J. L. Zhang , Z. H. Shi , J. Qi , Z. H. Zhang , D. Xue , J. Phys. Chem. C 2010, 114, 13477.

[42] L. Nian , W. Q. Zhang , N. Zhu , L. L. Liu , Z. Q. Xie , H. B. Wu , F. Würthner , Y. G. Ma , J. Am. Chem. Soc. 2015, 137, 6995. 26016386 10.1021/jacs.5b02168

[43] N. Sekine , C. H. Chou , L. K. Wei , Y. Yang , Org. Electron. 2009, 10, 1473.

[44] H. Y. Park , D. Lim , K. D. Kim , S. Y. Jang , J. Mater. Chem. A 2013, 1, 6327.

[45] D. H. Sin , S. B. Jo , S. G. Lee , H. Ko , M. Kim , H. Lee , K. Cho , ACS Appl. Mater. Interfaces 2017, 9, 18103. 28497686 10.1021/acsami.7b02349

[46] P. M. Pihko , Angew. Chem., Int. Ed. 2004, 43, 2062. 10.1002/anie.200301732 15083451

[47] J. P. M. Lommerse , S. L. Price , R. Taylor , J. Comput. Chem. 1997, 18, 757.

