# Supplementary material for: Highly Efficient Flexible Polymer Solar Cells with Robust Mechanical Stability
Source: Adv Sci (Weinh). 2019 Feb 7;6(7):1801180. doi: 10.1002/advs.201801180 (PMC6446608; doi:10.1002/advs.201801180)
Supplement: Supplementary file 1 — Supplementary [file ADVS-6-1801180-s002.pdf]

Copyright WILEY-VCH Verlag GmbH & Co. KGaA, 69469 Weinheim, Germany, 2019.

ADVANCED SCIENCE
Open Access

Supporting Information

for *Adv. Sci.,* DOI: 10.1002/advs.201801180

Highly Efficient Flexible Polymer Solar Cells with Robust Mechanical Stability

Licheng Tan, Yilin Wang, Jingwen Zhang, Shuqin Xiao, Huanyu Zhou, Yaowen Li, Yiwang Chen,* and Yongfang Li*

Supporting Information

Highly Efficient Flexible Polymer Solar Cells with Robust Mechanical Stability

Licheng Tan[1,2], Yilin Wang[1], Jingwen Zhang[3], Shuqin Xiao[1,2], Huanyu Zhou[1], Yaowen Li*[3], Yiwang Chen*[1,2], Yongfang Li[3]

[1]College of Chemistry, Nanchang University, 999 Xuefu Avenue, Nanchang 330031, China

[2]Institute of Polymers and Energy Chemistry, Nanchang University, 999 Xuefu Avenue, Nanchang 330031, China

[3]Laboratory of Advanced Optoelectronic Materials, College of Chemistry, Chemical Engineering and Materials Science, Soochow University, 199 Ren'ai Road, Suzhou 215123, China

Corresponding author. Tel.: +86 791 83968703; fax: +86 791 83969561. E-mail: ywchen@ncu.edu.cn (Y.W.C.), ywli@suda.edu.cn (Y.W.L.). Licheng Tan and Yilin Wang contributed equally.

Experimental Section

Materials and reagents: Patterned ITO glass substrates (sheet resistance ≤ 10 Ω/sq; transmittance ≥ 84%) were purchased from Zhuhai Kaivo Optoelectronic Technology Co. Ltd. PTB7-Th, PBDB-T and ITIC were purchased from 1-material, Chemscitech Inc., QC, Canada. $PC_{71}BM$ was purchased from Solarmer Materials Inc., China. Chlorobenzene (CB) and 1,8-diiodoctane (DIO) were obtained from Sigma-Aldrich. All available chemical reagents were used as received without any further purification.

Preparation of PDA solution. 0.09 g tris hydrochloride and 0.17 g trometamol were added into the 200 mL deionized water to prepare tris buffer. Then, 3-hydroxytyramine hydrochloride (J&K chemical) was dissolved in the tris buffer (pH 8.5). The dopamine monomer was self-aggregated at 25 ℃.

Preparation of AZO and AZO:PDA. The general procedure for the preparation of AZO solution according to the published literature.[1] In details, 2.195 g zinc acetate dihydrate ($Zn(CH_3COO)_2·2H_2O$, Aldrich, 98%) and doping source of 0.038 g aluminum nitrate ($Al(NO_3)_3·9H_2O$, Aladdin 99.99%) were added into anhydrous ethanol (25 mL) in a round-bottom flask. After stirring for 10 min at 80 °C, 0.61 mL ethanolamine (EA, Aladdin, 99.5%) was injected into the turbid reaction solution and the ratio of EA to $Zn(CH_3COO)_2·2H_2O$ was retained at 1.0. It is worth noting that the suspension dissolved with the addition of EA gradually. Transparent AZO solution was obtained after 2 h stir and reflux at 80 °C. For AZO:PDA hybrid solutions, 10 mg/mL PDA was added into the AZO solution with different molar fraction (1.0%, 1.5% and 2.0%). Desired concentration was obtained by diluting the AZO solution with anhydrous ethanol when used.

Device preparation. The inverted solar cells studied in this work are based on the following layer sequence: ITO/AZO ETLs/active layer/MoO_3/Ag (or Al). For flexible device, the device structure is PET/Ag-mesh/PH1000/PEIE/ETLs/active

layer/MoO_3/Al. The treated substrates were dried with a flowing nitrogen stream and further treated by atmospheric plasma treatment for 3 min. After that, AZO and AZO:PDA solutions were spin-coated onto the ITO substrates as ETLs and annealed in air at 140 °C for 20 min. Then active layer was deposited by spin-coating from a PBDB-T-2F:IT-4F (10:10 wt%, 20 mg/mL) solution in mixed solvents of CB:DIO (99.0:1.0 vol%) under 1600 rpm for 60 s dried on a hot plate at 100 °C for 15 min. For PBDB-T:ITIC system, the active layer was deposited by spin-coating from a PBDB-T:ITIC (10:10 wt%, 20 mg/mL) solution in mixed solvents of CB:DIO (99.5:0.5 vol%) under 2500 rpm for 60 s. For PTB7-Th:$PC_{71}BM$ system, the active layer was deposited by spin-coating from a PTB7-Th:$PC_{71}BM$ (6:9 wt%, 15 mg/mL) solution in mixed solvent of CB:DIO (97.0:3.0 vol%). Finally, MoO_3 and Ag (or Al) were deposited on top of the active layer by thermal evaporation in a high vacuum ($< 1 \times 10^{-6}$ Torr). For flexible device, the PEDOT layer (AI4083) was spin-coated onto PET/Ag-mesh electrode in ambient air and dried on a hot plate at 120 °C for 20 min. Then, PEIE is deposited on the PEDOT layer under 5000 rpm for 60 s.

Characterizations and device measurement. UV-Vis absorption and transmission spectra of AZO and AZO:PDA were taken with a Lambda 750 UV-Vis spectrometer. PL spectra of AZO and AZO:PDA were carried on a Hitachi F-7000 photoluminescence spectroscopy with a xenon lamp as the light source. The Philips X'Pert Pro MPD with Cu Kα radiation (λ = 1.54056 Å) was applied to gather XRD patterns under a scanning rate of 2 °/min. SEM images were taken by FEI XL30 Sirion SEM. Fourier transform infrared (FTIR) spectra were recorded on a Shimadzu IRPrestige-21 spectrometer. The surface morphologies of AZO and AZO:PDA were characterized by AFM (Digital Instrument Nanoscope 31). XPS was executed by employing a Kratos AXIS Ultra. UPS was carried out by the AXISULTRA DLD spectrometer (Kratos Analytical Ltd.). For the UPS measurements, He I (21.22 eV) radiation line from a discharge lamp was used, with an experimental resolution of 0.15 eV. All UPS measurements of the onset of photoemission for determining the work function were done using standard procedures with a -5 V bias applied to the

sample. J-V characteristics were tested using a Keithley 2400 sourcemeter under AM 1.5 G solar spectra illumination (100 mW/cm^2, Abet Solar simulator Sun2000). IPCE values were measured under monochromatic illumination (Oriel Cornerstone 260 1/4 m monochromator equipped with an Oriel 70613NS QTH lamp), and the calibration of the incident light was performed using a monocrystalline silicon diode. Mechanical bending test was measured by a stepper motor controller (CL-01A). For peeling test, we apply a force of 0.5 MPa to the 3M tape and active layer by tabletting machine. Subsequently, the sample is putted on the universal testing machine (WOTEI-20) for peeling test (2 N peeling force, 50 mm/min peeling speed and 180° peeling direction).

Energies level of ultraviolet photoelectron spectroscopy (UPS)

The lowest unoccupied molecular orbital (LUMO) energies were determined according to the equation:[2,3]

$$E_{LUMO} = h\nu - (E_{cutoff} - E_{onset}^{HOMO})$$

where hν is the incident photon energy (hν = 21.2 eV). As shown in **Figure S4**, the left panel is the E_{cutoff} gained from the high binding energy cutoff of a spectrum, and the right panel provides E_{onset}^{HOMO}, which is the onset relative to the Fermi level of Au (at 0 eV).

Space-charge-limited-current (SCLC) mobility measurement

In order to characterize the carrier mobility of modified device, electron-only devices with configuration of ITO/AZO ETLs/PBDB-T:ITIC/Al were fabricated The carrier mobility was measured using the Mott-Gurney SCLC model at low voltage which is described by the following equation:[4]

$$J = (9/8)\, \mu_e\, \varepsilon_0\, \varepsilon_r\, (V^2/L^3)$$

where J is the current density, μ_e is the effective charge carrier mobility which includes the effect of injection efficiency or traps, ε_0 is the permittivity of free-space, ε_r is the relative dielectric constant, V is the applied voltage, and L is the thickness of ETLs and active layers.

References

1. X. H. Liu, X. D. Li, Y. R. Li, C. J. Song, L. P. Zhu, W. J. Zhang, H. Q. Wang, J. F. Fang, Adv. Mater. **2016**, 28, 7405.
2. J. H. Seo, R. Q. Yang, J. Z. Brzezinski, B. Walker, G. C. Bazan, T. Q. Nguyen, Adv. Mater. **2009**, 21, 1006.
3. S. Braun, W. R. Salaneck, M. Fahlman, Adv. Mater. **2009**, 21, 1450.
4. H. Y Park, D. Lim, K. D. Kim, S. Y. Jang, J. Mater. Chem. A **2013**, 1, 6327.

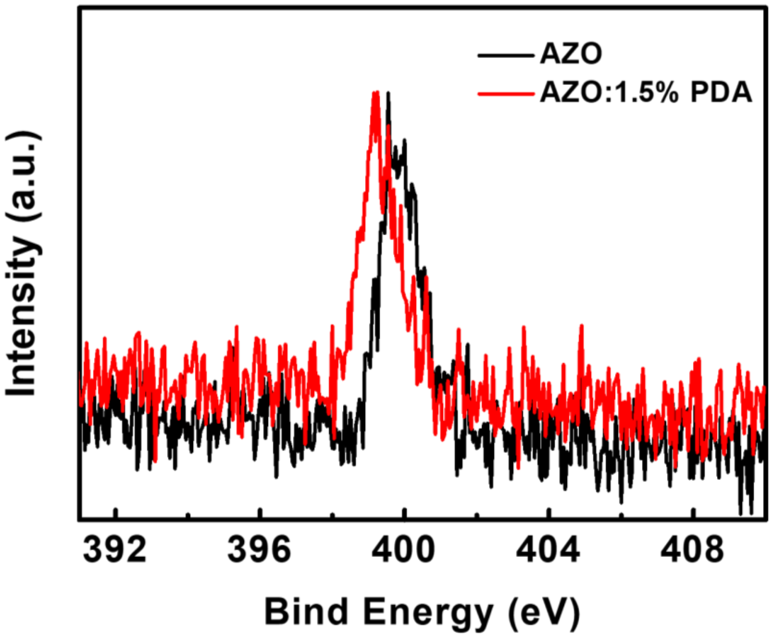

Figure S1. N 1s X-ray photoelectron spectroscopy (XPS) spectra of AZO and AZO:1.5%PDA.

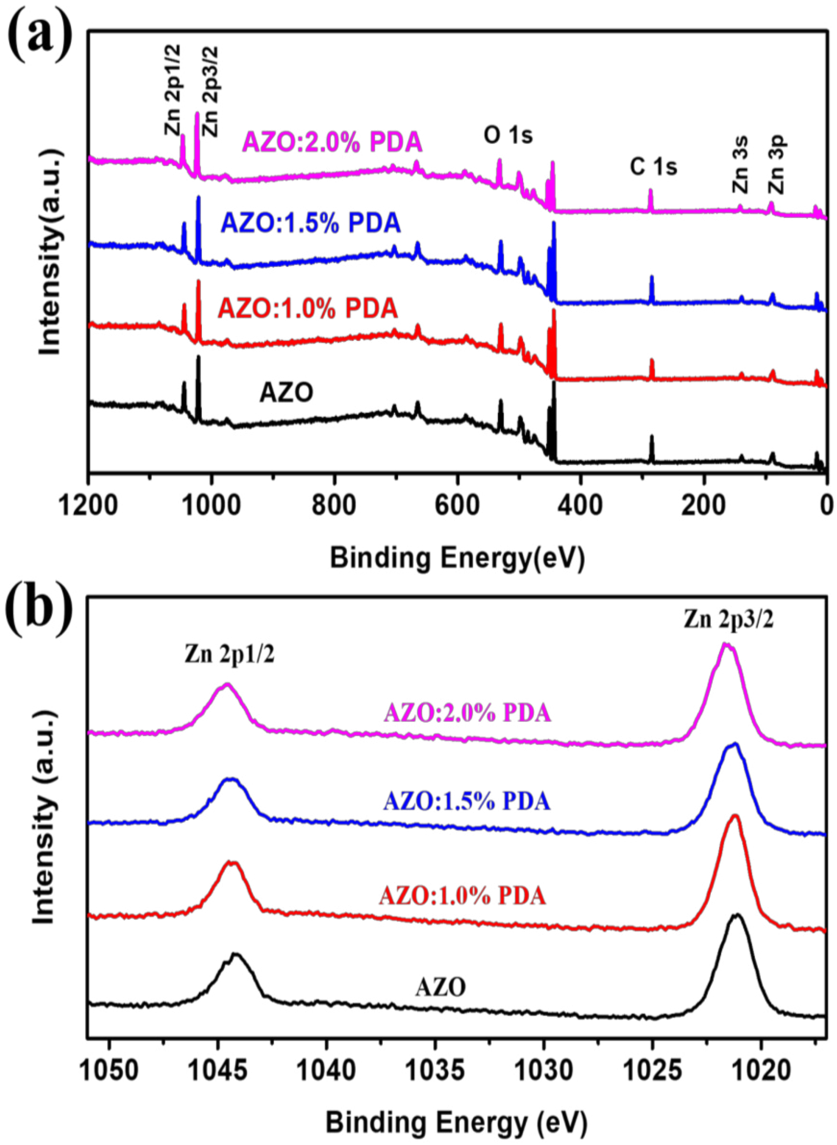

Figure S2. (a) XPS spectra of AZO, AZO:1.0%PDA, AZO:1.5%PDA and AZO:2.0%PDA. (b) Zn 2p of AZO, AZO:1.0% PDA, AZO:1.5%PDA and AZO:2.0%PDA.

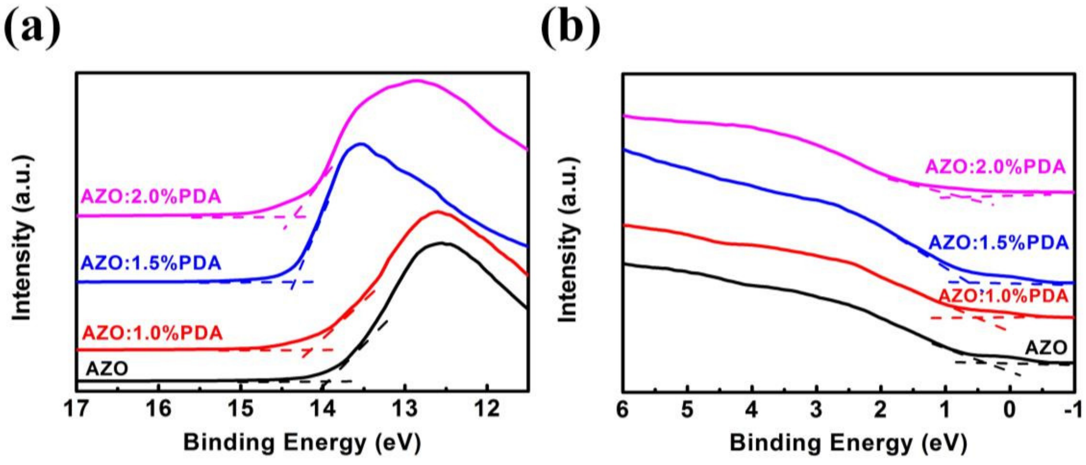

Figure S3. (a) Ultraviolet photoelectron spectroscopy (UPS) results of the corresponding energy range near the secondary electron cutoff and (b) Fermi/the highest occupied molecular orbital (HOMO) level.

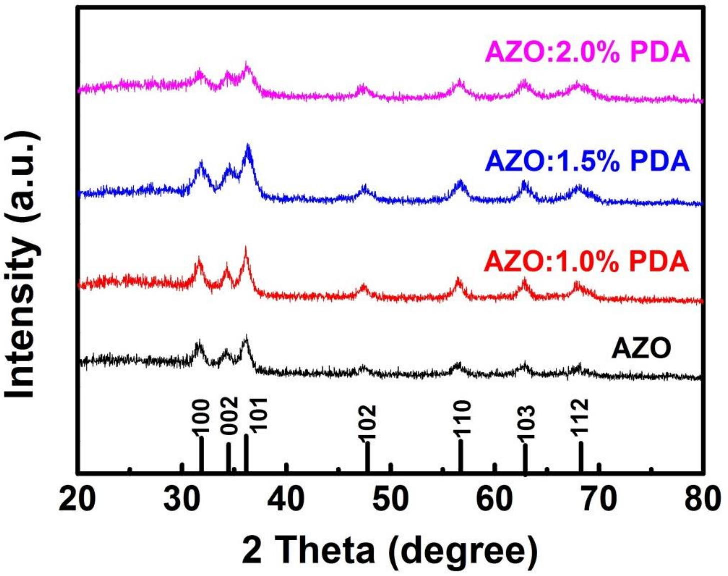

Figure S4. X-ray diffraction (XRD) patterns of AZO, AZO:1.0%PDA, AZO:1.5%PDA and AZO:2.0%PDA.

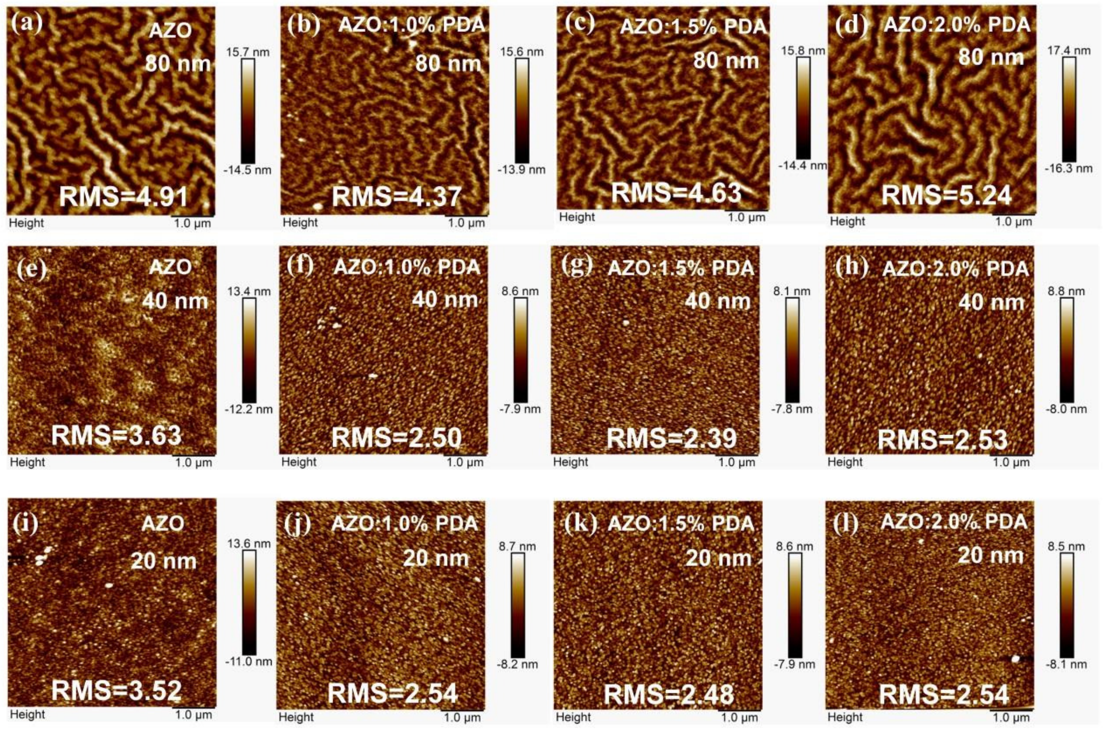

Figure S5. Atomic force microscopy (AFM) surface plot images (5 μm × 5 μm) of AZO, AZO:1.0%PDA, AZO:1.5%PDA and AZO:2.0%PDA films: (a-d) ~ 80 nm, (e-h) ~ 40 nm and (i-l) ~ 20 nm, respectively.

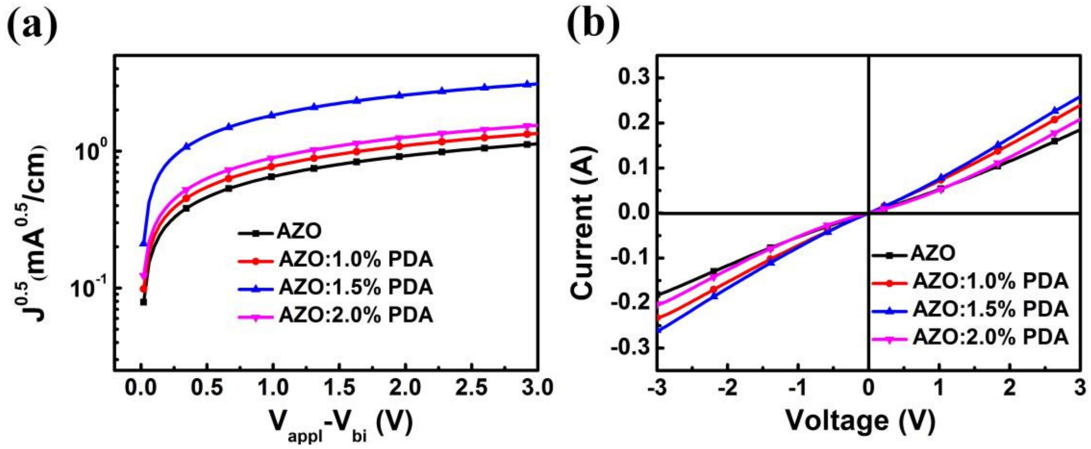

Figure S6. (a) Current density$^{0.5}$-voltage ($J^{0.5}$-V) curves of electron-only devices based on ITO/AZO ETLs/PBDB-T:ITIC/Al, (b) J-V curves of ITO/AZO ETLs/Al.

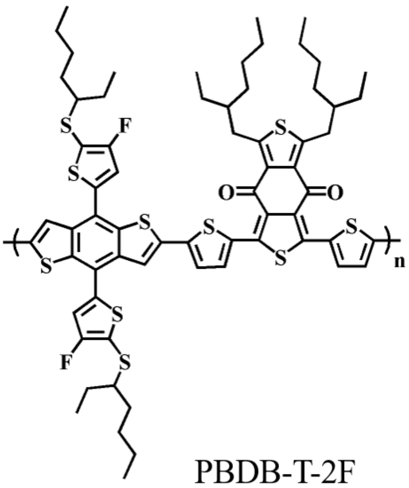

PBDB-T-2F

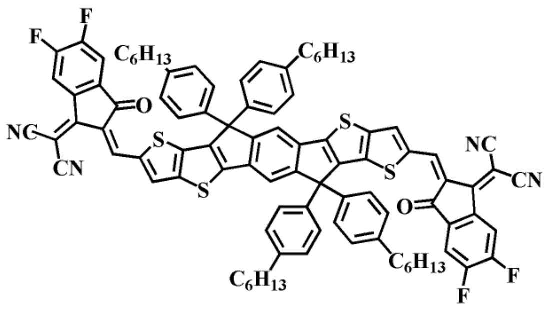

IT-4F

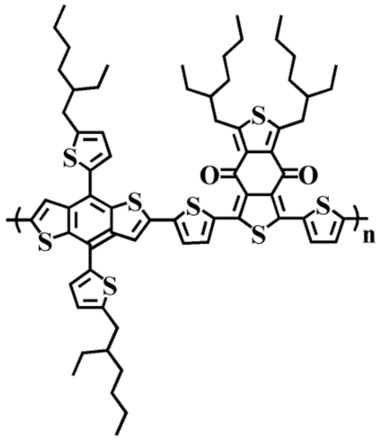

PBDB-T

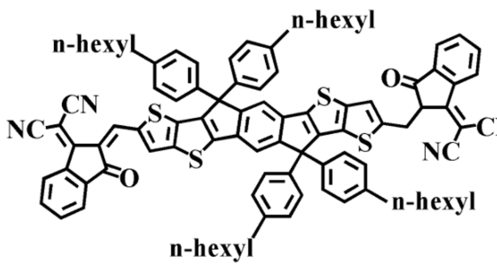

ITIC

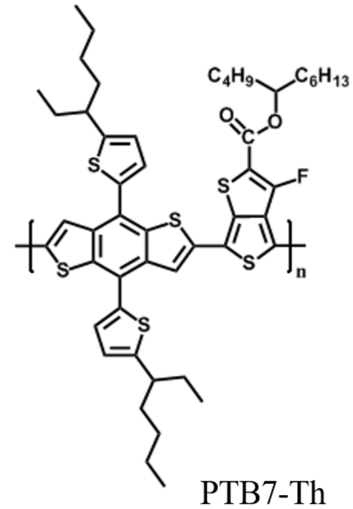

PTB7-Th

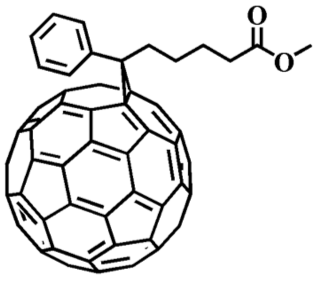

$PC_{71}BM$

Figure S7. The molecular structures of the active layer materials.

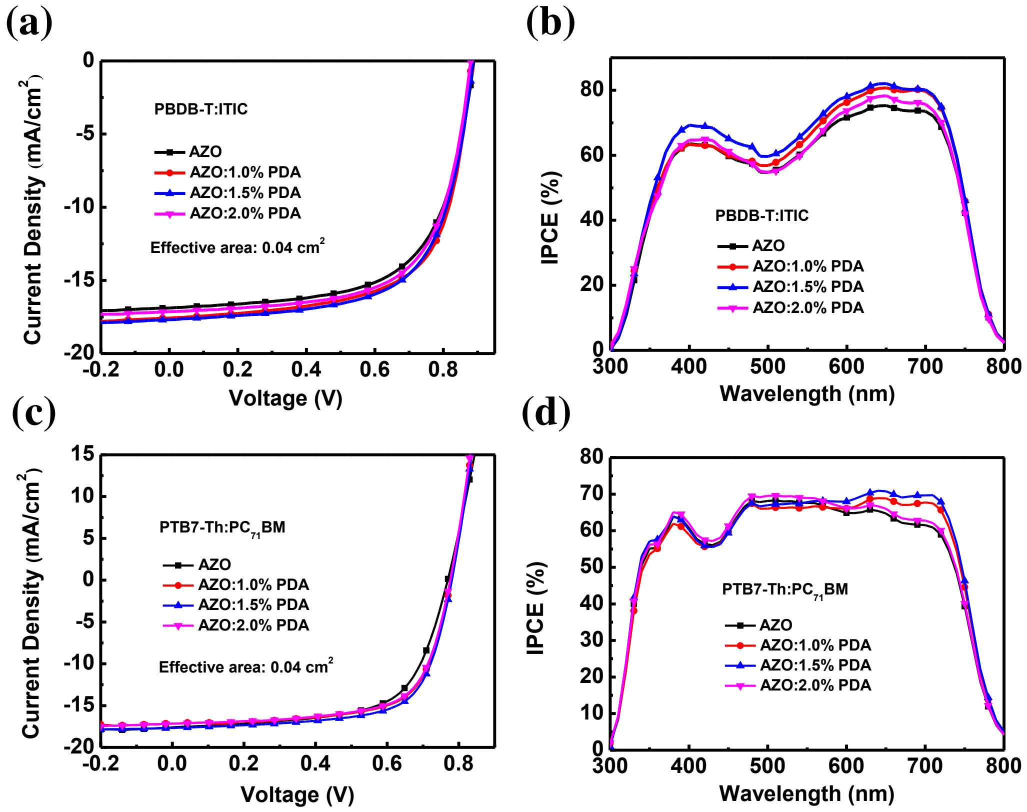

Figure S8. (a, c) The current density-voltage (J-V) curves and (b, d) incident photon-to-current efficiency (IPCE) spectra of the inverted PSCs based on ITO/ETLs/PBDB-T:ITIC/MoO_3/Ag and ITO/ETLs/PTB7-Th:$PC_{71}BM$/MoO_3/Ag, respectively.

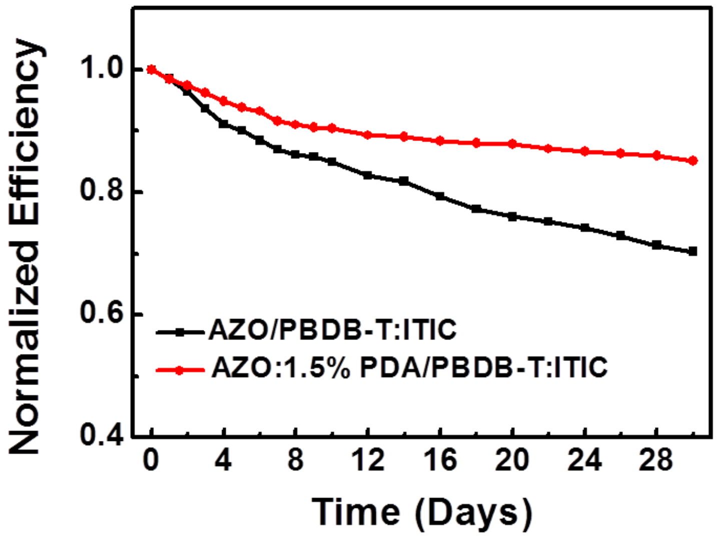

Figure S9. Comparison of the average power conversion efficiency (PCE) stability of AZO/PBDB-T:ITIC and AZO:1.5%PDA/PBDB-T:ITIC based PSCs without encapsulation.

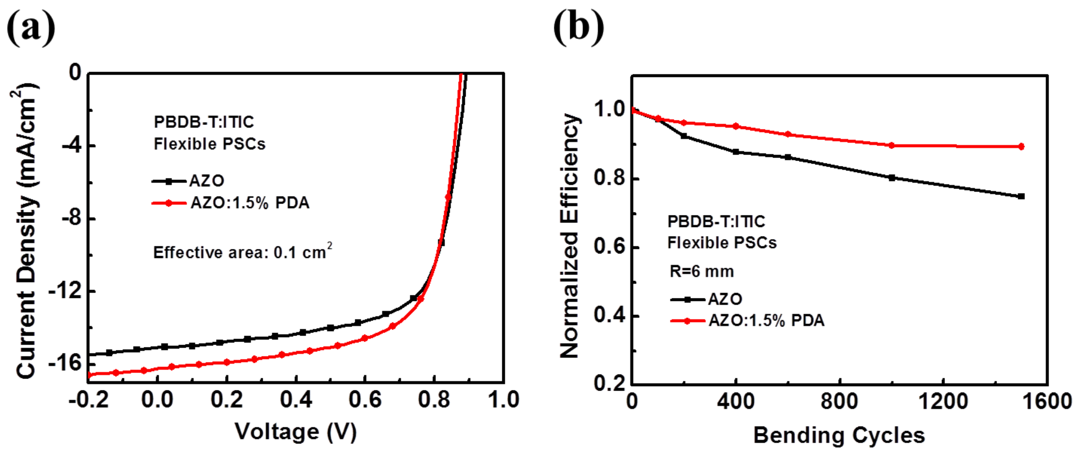

Figure S10. (a) J-V curves of flexible devices based on PET/Ag-mesh/PH1000/PEIE/AZO ETLs/PBDB-T-2F:IT-4F/MoO_3/Al. (b) Normalized average PCE of flexible PSCs as a function of bending cycles with radius of 6 mm.

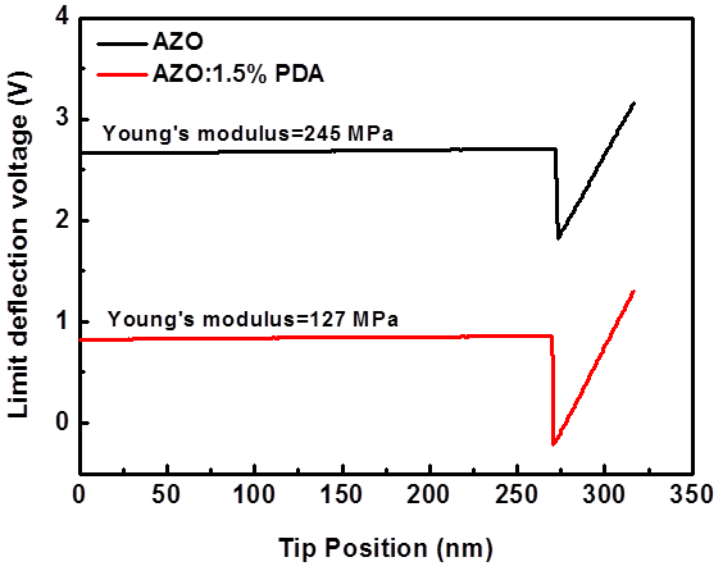

Figure S11. Young’s model measured by peak-force model of atomic force microscopy (AFM) of AZO and AZO:1.5%PDA.

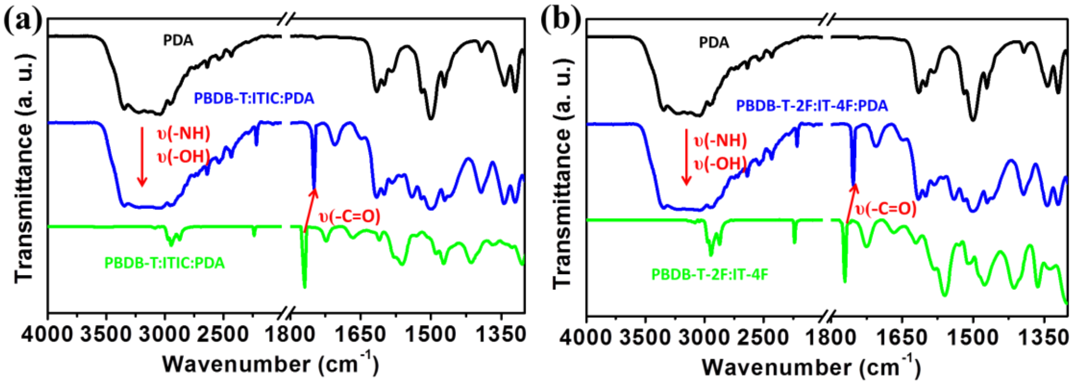

Figure S12. Fourier transform infrared spectroscopy (FTIR) spectra of a) PDA, PBDB-T:ITIC:PDA and PBDB-T:ITIC. b) PDA, PBDB-T-2F:IT-4F:PDA and PBDB-T-2F:IT-4F.

Table S1. Electron mobility of devices based on ITO/AZO ETLs/PBDB-T:ITIC/Al.

ETLs	AZO	AZO:1.0%PDA	AZO:1.5%PDA	AZO:2.0%PDA
Electron mobility [cm^{-2} V^{-1} s^{-1}]	6.1×10^{-4}	8.2×10^{-4}	4.2×10^{-3}	1.1×10^{-3}

Table S2. Conductivity of devices based on ITO/AZO ETLs/Al.

ETLs	AZO	AZO:1.0%PDA	AZO:1.5%PDA	AZO:2.0%PDA
Conductivity [S/m]	1.18×10^{-3}	1.54×10^{-3}	1.68×10^{-3}	1.28×10^{-3}

Table S3. Device parameters of inverted PSCs based on PBDB-T:ITIC and PTB7-Th:$PC_{71}BM$ blend with AZO and bendable AZO ETLs under AM 1.5G irradiation (100 mW cm^{-2}).

ETLs	BHJ	J_{sc} (mA/cm^2)	V_{oc} (V)	FF (%)	PCE (%)
AZO	PBDB-T:ITIC	16.81±0.18	0.880±0.002	62.5±1.2	9.5±0.2 (9.7)[a]
AZO:1.0%PDA	PBDB-T:ITIC	17.41±0.17	0.880±0.002	64.3±1.4	10.0±0.1 (10.1)[a]
AZO:1.5%PDA	PBDB-T:ITIC	17.54±0.14	0.882±0.004	64.3±1.0	10.1±0.2 (10.3)[a]
AZO:2.0%PDA	PBDB-T:ITIC	16.98±0.15	0.881±0.002	64.2±1.0	9.9±0.1 (10.0)[a]
AZO	PTB7-Th:$PC_{71}BM$	17.33±0.17	0.770±0.004	64.1±1.2	8.6±0.1 (8.7)[a]
AZO:1.0%PDA	PTB7-Th:$PC_{71}BM$	17.35±0.19	0.780±0.004	70.1±1.2	9.0±0.1 (9.1)[a]
AZO:1.5%PDA	PTB7-Th:$PC_{71}BM$	17.53±0.14	0.780±0.003	70.4±1.1	9.5±0.1 (9.6)[a]
AZO:2.0%PDA	PTB7-Th:$PC_{71}BM$	17.32±0.18	0.780±0.003	69.4±1.1	8.9±0.1 (9.0)[a]

The device effective area is 0.04 cm^2 of a single chip; all the values represent averages from twelve devices on a single chip.

The device structure is ITO/AZO ETLs/Active layer/MoO_3/Ag.

[a] The best PCE value.

Table S4. The decay of PCE for inverted PSCs based on PBDB-T-2F:IT-4F and PBDB-T:ITIC after bending test.

ETLs \ PCE(%) \ Bending cycles	**0**	**100**	**200**	**400**	**600**	**1000**	**1500**	**BHJ**
AZO	11.2	10.6	10.2	10.0	9.0	8.5	8.3	PBDB-T-2F:IT-4F
AZO:1.5%PDA	11.5	11.3	11.1	11.1	10.8	10.6	10.3	
AZO	9.2	8.9	8.5	8.0	7.9	7.4	6.9	PBDB-T:ITIC
AZO:1.5%PDA	9.6	9.4	9.3	9.2	8.9	8.6	8.6	

Movie S1. The video shows the 3M tape peeling test of ITO/AZO:1.5% PDA/PTB7-Th:$PC_{71}BM$ after 10 times.

Movie S2. The video shows the 3M tape peeling test of ITO/AZO/PTB7-Th:$PC_{71}BM$ after 5 times.

Movie S3. The video shows the 3M tape peeling test of ITO/AZO:1.5% PDA/PBDB-T:ITIC after 10 times.

Movie S4. The video shows the 3M tape peeling test of ITO/AZO/PBDB-T:ITIC after 5 times.
